# Progress in State Medicine

**Published:** 1901-10-05

**Authors:** 


### Progress in State Medicine.

### PLAGUE AND ITS PREVENTION.

Clemow[1] remarks that the association of epidemic disease in human beings with disease in the lower animals is as old as the history of epidemic disease itself. But this co-existence of disease in man and brute is most often and most definitely met with in the case of plague. On three separate occasions monkeys, living under natural conditions, have died from a disease resembling plague at the time of an epidemic of that disease in man. On each occasion the specific nature of the disease in these animals was proved bacteriologically. Under artificial conditions the monkey is very susceptible to the plague bacillus. There is little proof that they act to any considerable extent in spreading the disease, and there is no evidence that insect-eating mammals and bats ever become the host of the plague bacillus either under natural or artificial conditions. The rodentia are the most susceptible of all animals to plague. Rats when attacked with this disease usually leave their underground habitations and migrate often for a considerable distance. In the plague epidemic of 1898 in Calcutta the rats almost disappeared from the native quarters where they had before swarmed. A considerable mortality occurred among those remaining. They may be infected from (1) the soil, (2) grain or other foodstuffs, (3) tissues of other animals, including human beings dead from the plague, (4) infected rags or dressings from plague patients, (5) infected insects. It may be taken as proven that soil may, under certain circumstances, contain the virus of plague. Grain becomes infected by dejecta, sputa, or by the dead bodies of such as die among it. Whether a rat epizootic can be started by infected grain brought from a distance is uncertain. The risk which the plague cadaver offers as a means of infecting rats is recognised in most plague hospitals where a cover of wire or other material is provided to protect bodies. There is good reason to believe that plague is spread from rats to other rats by insects of which probably the flea is the most important. It is uncertain how the disease is transmitted to man from rats. Direct transmission is rare. The development of plague in persons who have actually handled a living or dead infected rat are more common. On no recent occasion has it been possible to trace an outbreak of plague to a definite introduction of the infection by sick or dead rats upon a certain known ship. Among other animals susceptible to plague are bandicoots, mice, squirrels, guinea-pigs, porcupines, and marmots. Crimmin and Targett[2] writing on the Sanitary Administration of the Port of Bombay, assert that before plague becomes established as an epidemic within an uninfected area much must depend upon many local factors, insanitary conditions, density of population and buildings, the quantity of infective material introduced, together with the frequency of its introduction, its vitality and virulence, as influenced by the means of transport from the infected area to the uninfected area, and its mode of introduction. For example, the infectivity of a Lascar's clothing will depend on their exposure to air, sunlight, moisture, warmth, or the contrary. Passengers on short voyages may complete their incubation period after leaving the ship, and thus carry the most active infection with them ashore. An infected rat may convey the infection, infecting by fleas or otherwise the shore rats. The transmission of plague from individual to individual is not readily established amongst Europeans in India, save in the pneumonic form. Its transmission appears to be chiefly, if not entirely, conveyed by means of direct inoculation, not excepting the so-called pneumonic type. Other circumstances which materially increase, alter, and modify the risks of the propagation of plague among Oriental people are religion, caste, prejudices, habits, and customs. All passengers leaving Bombay by sea are medically examined prior to embarkation. Members of the crew are likewise treated, and the bedding and clothing of Asiatics (with the exception of Japanese) and African members of the crew disinfected. Native deck passengers after disinfection of their baggage and careful medical inspection, have their forearm stamped with a rubber stamp, bearing the date on which the examination took place so as to prevent an unexamined passenger taking the place of one who has passed the examination. Hospitals and isolation camps are provided on shore for certified and suspicious cases. The greatest difficulty to be contended with in India is the corrupt character of all the native overseers. The Indian Plague Commission reported that little danger is to be apprehended by the spread of plague by ordinary articles of merchandise, and that it is neither wise nor necessary to take any steps towards disinfection of ordinary merchandise. Further, the Commissioners state that although theoretically it is possible that plague infected rats might carry the disease from one country to another, there is no evidence that infection has ever been carried in this manner. They think also that the suggested fumigation of the holds of ships so as to destroy the rats is both unnecessary and impracticable. Davies[3] points out that although Cantlie in 1895 ventured the pregnant supposition that the endemicity of plague in certain parts of Central Asia was possibly in causal relationship with the defined rat habitat, it was Manson who forcibly insisted upon the necessity for dealing with plague as essentially a rat-borne disease. He is of opinion that the records of the Sydney outbreak of plague in 1900 confirm in every particular that plague in man is a secondary event dependent on previous plague in animals. His grounds for this are that the disease did not spread by person-to-person infection; its spread can only be explained by assuming an animated host, not human, to diffuse the infection in place, and a second host to communicate the infection to man; the two hosts are the rat and the flea. Nearly all the cases were bubonic in type, a type very little infectious. Further, the outbreak originated in connection with a wharf, almost certainly through the landing of plague rats from infected ports. He asks whether our present regulations are sufficient to prevent the introduction of plague, or are the measures we are empowered to take against it most accurately adapted to its special nature and its etiology. He alludes to the arrival in Bristol of a grain boat from Smyrna having no sickness on board. The cargo had been partly discharged when thirteen dead rats were found in the forehold, certain of which Professor Klein confirmed had died from plague. Thereupon the ship was dealt with as an "infected" ship, and no case of plague resulted. Disinfection was in this case sufficient, but more is required. Merchant vessels should take the necessary steps for the destruction of rats on board before loading and upon discharge, and also to prevent intercommunication during loading and discharge between ship and shore rats. Further,[4] he counsels owners of riverside warehouses and granaries to make systematic efforts to exterminate rats; to use cables with special conical guarded collars, raise footbridges during the night. Fumigation with sulphurous acid gas effectually destroys rats in a closed hold, but this can only be used after the hold is cleared out. A capitation grant of a penny per rat would ensure the destruction of half a million rats. Any rats found on a "suspected" vessel must not be handled, but plunged with tongs into a bucket of carbolic solution until they can be burnt. No rats must be thrown overboard into the harbour. A trial[5] made in the London docks of flooding the hold of a vessel with sulphurous dioxide gas for destroying rats was completely successful. The gas was pumped on board in the afternoon, all ventilating apertures and other openings having been closed. On opening the ship on the following day a considerable number of rats were found dead in various situations. The animals were quite dead and no fleas could be observed about them. The time taken in filling the vessel will ultimately, with improved machinery, be considerably reduced. Experiments[6] have also been continued with Danysz's bacillus for the extermination of the rat. Its pathogenicity for rats and mice is confirmed, but it is found that after three or four passages through rats the bacillus begins to lose virulence, after ten such passages its virulence has disappeared. It is without effect upon birds, cats, dogs, and guinea-pigs. When a rat is allowed to eat bread soaked in a culture of the bacillus, and placed in a cage containing other rats, upon its death the carcase is eaten by the other rats, who then die with lesions typical of the disease. The lesions produced by the bacillus in rats and mice were those of a septicæmia with marked engorgement of the spleen; the glandular affections produced by the B. pestis were absent. The difficulty of its application on a large scale will be to find a method of enhancing and maintaining the virulence of the laboratory cultures. Doty[7] is of opinion that many of the methods connected with quarantine are not in harmony with scientific investigation and practical experience. The term "commerce destroyer" which has been applied to quarantine has not been used without some justification. Knowledge of germ origin, and germicidal agents known as disinfectants leaves little reason for continuation of some of the methods now employed. Consideration should be taken that ships' cargo and clothing of healthy people are not sources of infection. Whilst agreeing that rats may be a source of infection in some of the Asiatic ports, he thinks that in civilised communities, where the ordinary sanitary regulations are carried out, the danger from this source would be very limited. No authentic report exists which shows that cargoes of vessels have transmitted bubonic plague through the medium of infected rats or other sources. For 30 years ships trading between infected Asiatic ports and America, carrying raisins, rugs, silks and other general merchandise, have been restricted by no quarantine regulations, and in no instance has the disease been conveyed into America. During the outbreak of plague in Rio Janeiro and Santos many vessels from these ports were unloaded in mid-stream at the New York quarantine station, and all rats dead or alive collected. Bacteriological examination failed to find the slightest evidence of plague infection in every case. It is the mild or ambulant cases of infectious disease, and convalescents from these diseases which constitute a menace to the public health and not infected rags, cargo or rats. At the New York quarantine station all passengers and crews coming from a plague-infected port are not released until their temperatures have been taken. All those whose temperature is 100° Fahr. or over are detained.

[1] Brit. Med. Jour., May 12, 1900. [2] Public Health, Aug. 1901. [3] Brit. Med. Jour., Aug. 10, 1901. [4] Rep. M.O.H., Bristol Port Sanitary Authority. 1900. [5] Brit. Med. Jour., May 11, 1901. [6] Brit. Med. Jour., May 18, 1901. [7] Med. Rec., Nov. 3, 1900.

